# Reach and Effectiveness of a Weight Loss Intervention in Patients With Prediabetes in Colorado

**Published:** 2010-08-15

**Authors:** Fabio A. Almeida, Susan Shetterly, Renae L. Smith-Ray, Paul A. Estabrooks

**Affiliations:** Virginia Polytechnic Institute and State University, Human Nutrition, Foods, and Exercise. When this research was conducted, Dr Almeida was affiliated with the Institute for Health Research, Kaiser Permanente Colorado, Denver, Colorado; Kaiser Permanente Colorado, Denver, Colorado; University of Illinois at Chicago, Chicago, Illinois. When this research was conducted, Ms Smith-Ray was affiliated with Kaiser Permanente Colorado, Denver, Colorado; Virginia Polytechnic Institute and State University, Roanoke, Virginia, and Kaiser Permanente Colorado, Denver, Colorado

## Abstract

**Introduction:**

Although lifestyle interventions are effective in delaying the onset of diabetes, translating these lessons to routine health care settings remains a challenge. We investigated the effectiveness of a theory-based, brief, small-group weight loss intervention for diabetes prevention. A secondary purpose was to determine the potential reach of the intervention.

**Methods:**

A total of 14,379 members of an integrated health care organization newly diagnosed with prediabetes were potentially eligible to participate in this matched cohort longitudinal study. Of this group, 1,030 attended a 90-minute, small-group session that targeted personal action planning for healthful eating, physical activity, and weight management. We accessed electronic medical records to select 1 to 2 controls (matched on impaired fasting glucose measurement, sex, age, and body mass index) for each member who attended the small-group session (n = 760). Weight change, as recorded in the medical record, was the primary outcome. Mixed models analyses were used to adjust for matching variables and covariates and to account for individual random effects over time.

**Results:**

Small-group participants lost significantly more weight than did their matched controls. A significantly higher proportion of small-group participants lost at least 5% of their body weight compared with controls.

**Conclusion:**

A brief, small-group weight loss intervention was effective. However, it did not reach broadly into the population that was at risk for diabetes.

## Introduction

Complications related to uncontrolled diabetes include blindness, end-stage renal disease, high blood pressure, nervous system damage, dental disease, and complications of pregnancy (1). No known treatment is available to cure diabetes, and self-management for those with diabetes remains a challenge; therefore, prevention is paramount (1,2). Healthy lifestyle changes such as eating a healthful diet and increasing physical activity levels have been effective for preventing the onset of type 2 diabetes (3,4). The Diabetes Prevention Program (DPP), a large multisite clinical trial of interventions to delay or prevent the onset of type 2 diabetes among overweight and obese adults with prediabetes (3), found that, although some medications may help delay the development of diabetes, there was a 58% reduction in the incidence of diabetes resulting from 30 minutes of physical activity per day 5 times a week coupled with a 5% to 10% weight loss (3).

Evidence continues to mount of the effectiveness of modest weight loss through lifestyle changes to delay the onset of type 2 diabetes and its complications (3-6). However, translating these lessons to routine health care settings with more modest resources remains a challenge. In published efficacy trials, most interventions are delivered with a high frequency of time-intensive sessions over a long time and need many resources (6). In DPP, for instance, each participant received 16 one-on-one visits with a health care provider addressing diet, physical activity, and plans for behavior modification, followed by monthly individual and group sessions and regular group physical activity sessions (3). A single health educator working with 4 physicians who cared for 4,000 patients each would need more than 50 hours per week during a 4-year period just to provide the one-on-one counseling sessions for their patients, leaving no time for the group or physical activity sessions (7). The question remains, how can health care settings with modest resources implement effective weight loss programs that help prevent or postpone type 2 diabetes?

An alternative may be less resource-intensive interventions, which are based on theoretical components of effective programs, developed by using a patient-centered approach and delivered through an integrated system. We developed a brief, theory-based weight loss intervention for diabetes prevention that used the content in DPP and applied it to a closed-group health care system, where it was offered to all patients with prediabetes. Our purposes were 1) to determine whether the brief intervention strategy could produce objectively assessed weight loss among patients newly diagnosed with prediabetes and 2) to determine the potential reach of the brief intervention strategy into the intended audience of patients with prediabetes. We hypothesized that a diabetes prevention class would have a small but positive effect on weight reduction.

## Methods

Kaiser Permanente Colorado (KPCO) is an integrated health care organization that primarily serves the greater Denver metropolitan area. Members who had newly diagnosed prediabetes were potentially eligible to participate in this matched-cohort longitudinal study. In 2006 we began to access existing electronic medical records dated from February 2004 through March 2005 to create a cohort of 14,379 people with impaired fasting glucose (IFG) measurements of 100 to 125 mg/dL. IFG values were chosen instead of a diagnosis of prediabetes because of high variability in the use of the prediabetes code and changes in its definition over time. The inclusion criteria for this study was having an IFG measurement of 100 to 125 mg/dL, being aged 18 years or older, and being a member of the health care organization for at least 6 months before the study start date of February 2004. We excluded people with 1) an IFG measurement of 126 mg/dL or higher, 2) a diabetes diagnosis in the first 30 days after the IFG measurement, and 3) a dietitian contact in the 6 months before the study period (Figure).

**Figure.**
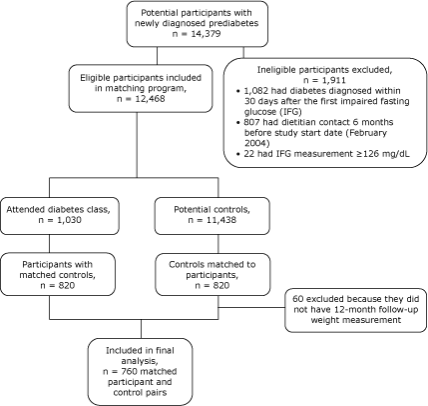
Flow of study participants in an intervention for patients with prediabetes, Kaiser Permanente Colorado, Colorado, 2004-2005. Dietitian contact refers to a potential participant having had an individual consultation with a dietitian and the reason coded as prediabetes.

Of the 12,468 eligible participants, 1,030 attended a single 90-minute small-group session that targeted personal action planning for healthful eating, physical activity, and weight management. This intervention, which was developed in partnership with KPCO, and its development process have been described in detail elsewhere (8). Briefly, each class of approximately 10 to 20 participants began with a presentation by a dietitian or weight loss specialist that included information about prediabetes and diabetes, recommendations for a healthful diet and regular physical activity, and information on how diet, physical activity, and weight loss delay the onset of diabetes. The 90-minute session, which was designed to incorporate social cognitive factors such as increasing self-efficacy, reducing barriers to physical activity, and identifying rewards for a healthful lifestyle, involved a question-and-answer period and small-group problem solving. At the conclusion of the session, participants created a personal action plan for preventing diabetes. The personal action plans were structured to include individual physical activity, healthful eating, weight loss goals, personal reasons for wanting to avoid diabetes (eg, maintain health in order to spend more enjoyable time with grandchildren), and strategies for decreasing barriers to the physical activity and dietary goals. Four to 6 classes were offered monthly from March 2004 through February 2005. These classes were advertised through KPCO's magazine with all health promotion offerings, KPCO's Web site, and doctor referrals.

We used electronic medical records to select 1 or 2 controls for each member who attended a small-group session. Matching was based on the following: exact match on month and year of IFG measurement, exact match on sex, within 5 years of age, within 2 body mass index (BMI) units, and within 5 mg/dL for initial IFG measurement. Each matched control was assigned a dummy date for participation in a small-group session; the date was identical to the date for its matched case. Matching was completed by using the first IFG result within the study time frame. People with a second IFG measurement before attending class or before their dummy small-group participation date had to have results in the IFG measurement range to ensure eligibility; otherwise, they were excluded from this study.

Weight, as recorded in the electronic medical records from February 2004 through April 2006, was used as the primary outcome to investigate the effectiveness of the intervention. Weight was measured by clinical staff using a weight scale during routine medical visits at that time. The weight measurement obtained closest to the date of participation in the small-group session (recorded up to 30 days before the session) was used as the baseline value for small-group participants; for matched controls, the weight recorded closest to the dummy participation date was used. Follow-up values were measurements made 12 months after initial class attendance (recorded up to 30 days after the end of the 12-month period). We eliminated 60 matched controls because of the lack of 12-month follow-up weight measurements that were within the 30-day window. This gave us 760 matched pairs for the final analyses (n = 1,520; mean age [SD], 63 [10]; 53% were women) (Figure). Participants in the small-group sessions (n = 760) were a mean age of 62 years. Racial and ethnic characteristic data were not available from the medical records used in this study. To determine the potential reach of the intervention, the proportional participation in the small-group sessions was calculated.

Although conditional analyses are typically used for studies that match on outcomes, it is less critical for studies that match on "exposure," as in this study (9). Mixed models analyses were used to adjust for matching variables and covariates and to account for individual random effects over time. Additionally, the percentage of participants losing a clinically significant amount of weight (≥5%) was calculated. To test for group differences between small-group participants and matched controls, the nonparametric χ^2^ test of independence was used. All tests were 2-sided with significance set at α = .05. The SAS software program version 9.1.3 (SAS Institute, Inc, Cary, North Carolina) was used to analyze the data. This study was approved by KPCO's institutional review board.

## Results

Of the 12,468 members eligible for participation in the small-group sessions, 1,030 took part, representing a participation rate of approximately 8% (Figure). Participants in the small-group sessions (N = 760) had an average weight of 188.3 lbs and average BMI of 29.8 kg/m^2^. χ^2^ And *t* test results confirmed that small-group participants did not significantly differ from their matched controls in demographic or weight attributes (Table).

Body weight for small-group participants measured 12 months after the start of the intervention decreased significantly more than that for their matched controls (mean weight loss for small-group participants, −3.0 lbs; 95% confidence interval [CI], −3.6 to −2.4; for controls, −1.4 lbs, 95% CI, −2.0 to −0.8; *P* < .001). When compared with their matched controls and adjusting for matching variables and initial weight, a significantly higher proportion of small-group participants lost at least 5% of their body weight (22% vs 15%, *P* = .001). Small-group participants were 1.5 (95% CI, 1.2-2.0) times more likely to lose at least 5% of their body weight than their matched controls.

## Discussion

Despite the growing evidence of the effectiveness of lifestyle changes in curbing and helping to avoid the onset of diabetes in patients (3-6), translating this knowledge into effective programs that can be delivered in routine health care settings remains a challenge. Our results confirm our hypothesis that a brief, small-group session on weight loss that is based on theoretical components of effective programs, developed using a patient-centered approach and delivered through an integrated system, can produce small but positive reductions in weight among patients newly diagnosed with prediabetes and can be delivered in health care settings. These results also support recent findings of a randomized controlled trial in Australia in a community setting, which found that a single-session health education program led to weight loss in young mothers and, more importantly, was as effective as a more resource-intensive, 4-session program (10).

Although the amount of weight loss was modest at 12 months after the initial delivery of the program, this is an important finding given the need for programs to show long-term results. In addition, approximately 1 in 5 participants lost at least 5% of their initial body weight — a clinically significant amount (3). From a public health perspective, a small improvement across a population at risk could have a large effect (11). We also found that the program had limited reach into the target population. This could be due to several factors, including insufficient class offerings, lack of awareness of the program, or patient time constraints. Extrapolating the findings by using the proportion of eligible patients that participated (approximately 1 in 10) and those who benefitted in a clinically meaningful way (1 in 5 participants), approximately 2% of the overall target population lost enough weight to delay the onset of diabetes. Unfortunately, trials such as DPP (3) do not provide concise information on program reach relative to a target population with a defined denominator, making comparisons at the population level impossible.

There are limitations to our study. First, participants were not randomly assigned to the small-group treatment; therefore, these results could be because members who were more motivated to lose weight chose to participate in the program. However, the matched cohort design allows for comparisons of patients with similar demographic and health profiles. Second, weight was not measured by trained research staff but by the staff of the integrated health care system. Still, no evidence shows that measures would be systematically different for patients who attended classes compared with those who did not. Finally, how these findings would or would not be generalizable to settings outside of an integrated health care system is unclear.

We conclude that a single-session, theory-based weight-loss program can be modestly effective but may not have sufficient reach to be effective as a population approach. This intervention was far less resource-intensive than DPP (3), and recent findings suggest that a brief program followed by interactive technology support could increase the magnitude of weight loss among participants without increasing resource cost (7). Therefore, a potentially fruitful area of future research would be to consider the cost-effectiveness of brief small-group interventions in comparative effectiveness trials with more intensive interventions and with interventions supplemented with follow-up through interactive technology.

## Figures and Tables

**Table.** Characteristics of Study Participants at Baseline in an Intervention for Patients With Prediabetes, Kaiser Permanente Colorado, Colorado, 2004-2005

**Participant Characteristic**	Intervention, n = 760	Control, n = 760	Overall, n = 1,520	*P* Value
Age, y, mean (SD)	62.4 (10.3)	62.6 (10.4)	62.5 (10.4)	.71^a^
Women, %	52.6	52.6	52.6	NA^a^
Initial mean weight, lb (SD)	188.3 (36.2)	187.7 (36.9)	188.0 (36.5)	.73^a^
Mean BMI, kg/m^2^ (SD)	29.8 (4.8)	29.8 (4.8)	29.8 (4.8)	.83^a^
Mean first IFG measurement, mg/dL (SD)	109.3 (6.2)	109.1 (6.2)	109.2 (6.2)	.48^a^
No. with second IFG measurement (%)	244 (32.1)	191 (25.1)	435 (28.6)	.002^b^
Mean second IFG measurement, mg/dL (SD)	109.2 (6.8)	107.7 (6.0)	108.5 (6.5)	.02^b^

Abbreviations: SD, standard deviation; NA, not applicable; BMI, body mass index; IFG, impaired fasting glucose.

a *P* values from *t* test are provided for matching variables that used a range for the matching. Sex was an exact match.

b Second IFG measurements were not matched. *P* values are based on χ^2^ test for percentage with a second measurement and *t* test for the second IFG measurement.
